# Decomposition of Organic Chemical Components in Wood by Tropical *Xylaria* Species

**DOI:** 10.3390/jof6040186

**Published:** 2020-09-23

**Authors:** Takashi Osono

**Affiliations:** Faculty of Science and Engineering, Doshisha University, Kyotanabe, Kyoto 610-0394, Japan; tosono@mail.doshisha.ac.jp

**Keywords:** acid unhydrolyzable residue, carbohydrate, Klason lignin, litter, selective delignification

## Abstract

The ability of *Xylaria* species obtained from tropical wood and leaf litter to cause a mass loss of lignin and carbohydrates in wood was examined in vitro with pure culture decomposition tests. The mass loss of wood of four tree species caused by nine *Xylaria* isolates ranged from 4.5% to 28.4% of the original wood mass. These *Xylaria* isolates have a potential ability to decompose lignin and other recalcitrant compounds, collectively registered as acid unhydrolyzable residues or Klason lignin in wood. The origin of isolates (i.e., isolates from wood versus leaf litter) did not affect the mass loss of acid unhydrolyzable residue in wood. The *Xylaria* isolates tested generally caused a selective decomposition of polymer carbohydrates in wood in preference to acid unhydrolyzable residue. The mass loss of acid unhydrolyzable residue caused by *Xylaria* isolates varied with the tree species of the wood and was negatively related to the initial content of acid unhydrolyzable residue in wood, implying the limiting effect of lignin and recalcitrant compounds on wood decomposition by *Xylaria* isolates.

## 1. Introduction

Fungi play critical roles in the decomposition processes of plant litter and in the mineralization of organic chemical components in forest ecosystems [1,2]. Their hyphae produce extracellular enzymes to decompose the lignocellulose matrix in the litter that is recalcitrant and often limits the decomposition of litter [3]. Fungi belonging to members of Basidiomycota and xylariaceous Ascomycota are of particular importance, as these are able to decompose the lignocellulose matrix [3]. The capabilities of individual fungal species in these taxa to decompose litter and recalcitrant organic chemical components have been examined with pure culture decomposition tests [4]. Quantifying these capabilities is crucial because such information highlights the roles of fungi as the dominant drivers of plant litter decomposition.

Fungi in the genus *Xylaria* (Xylariaceae, Sordariomycetes, Ascomycota) are known as ligninolytic to cause soft-rot type decomposition [5,6]. The ability of *Xylaria* species to cause a mass loss of wood and leaf litter has been examined under the pure culture, using isolates obtained from both the wood and leaf litter of temperate and tropical forests [7,8,9,10,11,12]. Moreover, data are accumulating on the ability of *Xylaria* species to decompose organic chemical components, such as lignin and polymer carbohydrates, in leaf litter [4]. In contrast, information is limited on the potential capabilities of *Xylaria* species to decompose organic chemical components in wood [7]. To the knowledge of the author, no data have been available regarding the decomposition of organic chemical components in wood by tropical *Xylaria* species, despite their importance as a major component of decomposer communities in tropical forests [6].

The purpose of the present study was to quantify the in vitro ability of nine *Xylaria* isolates obtained from tropical wood and leaf litter to cause a mass loss of Klason lignin (acid unhydrolyzable residue, AUR) and carbohydrates in wood. The AUR fraction contains a mixture of organic compounds in various proportions, including condensed tannins, phenolic and carboxylic compounds, alkyl compounds such as cutins, and true lignin [13]. In my previous study [11], wood, lamina, and petiole of tropical trees were inoculated with *Xylaria* isolates from tropical forests in northern Thailand during laboratory incubation to examine the ability of *Xylaria* isolates to cause a mass loss of these substrata. The wood samples of four tree species decomposed by these *Xylaria* isolates were used in the present study for the analysis of organic chemical components to illustrate the decomposition of lignin and carbohydrates caused by *Xylaria* isolates. A further aim was to estimate the possible effects of chemical composition of wood on fungal decomposition.

## 2. Materials and Methods

### 2.1. Fungal Isolates and Wood Materials

Nine *Xylaria* isolates were used in this study: five from wood and four from leaf litter (Table 1). These isolates were collected in October and November 2004 in northern Thailand and obtained from mass ascospores discharged from fruiting bodies collected from the twigs, branches, or logs, or petioles or primary veins of tree leaves or isolated from leaves. The isolates were identified taxonomically based on morphological observations and barcoding of the base sequences of the rDNA ITS region that were deposited in the DNA Data Bank of Japan (DDBJ) (Table 1). I refer to *Xylaria* isolates obtained from woody substrata (twigs, branches, and logs) as wood isolates and those from leaf litter as litter isolates. The terms wood and litter isolate do not indicate that these isolates are representatives of *Xylaria* species inhabiting or fruiting on wood and litter, respectively. In fact, molecular phylogenetic analysis showed that TP041001 from twig and TP5BS101 isolated from lamina have identical base sequences of their ITS region (Table 1), and thus the distinction between the wood and litter isolates is tentative. A total of 10 *Xylaria* isolates were used in the previous study [11], but in the present study I did not perform chemical analyses on wood samples inoculated with one litter isolate (TC041105) because said isolate caused a negligible mass loss of wood.

The wood of four tropical tree species (*Shorea obtusa*, *Dipterocarpus tuberculatus*, *Quercus kingiana*, and *Tectona grandis*) from northern Thailand were used as substrata in the pure culture test (Table 2). Wood blocks (approximately 10 × 10 × 5 mm) were cut out from living trees. Details of the collection of fungal isolates and substrata and a part of the results for the mass loss of wood have been described previously [11].

### 2.2. Pure Culture Decomposition Test

The method of pure culture decomposition tests has been described previously [11]. Samples of wood (approximately 400 mg) were sterilized by exposure to ethylene oxide gas at 60 °C for 6 h. The sterilized wood was placed on the surface of Petri dishes (9 cm diameter) containing 20 mL of 2% agar medium. Inocula for each assessment were cut out of the margin of previously inoculated Petri dishes on 1% malt extract agar medium (malt extract 1% and agar 2% (*w*/*v*)) and placed on the agar medium adjacent to wood. The plates were incubated for 12 weeks at 20 °C in the dark. The plates were sealed firmly with laboratory film during incubation so that moisture did not limit decomposition on the agar medium. After incubation, the wood was retrieved, oven-dried at 40 °C for 1 week, and weighed. The initial, undecomposed materials were also sterilized, oven-dried again at 40 °C for 1 week, and weighed to determine the original mass. Four plates were prepared for each isolate and each substratum, and four uninoculated plates served as a control for each substratum. The mass loss of wood was determined as a percentage of the original mass, taking the mass loss of materials in the uninoculated and incubated control treatment into consideration, and the mean values were calculated for each isolate and each substratum. Prior to the tests, the sterilized substrata were placed on 1% malt extract agar medium, and after 8 weeks of incubation at 20 °C in darkness, no microbial colonies had developed on the plates. This confirmed the effectiveness of the sterilization method used.

### 2.3. Chemical Analysis

Replicate wood samples were combined to make one sample for each assessment and ground in a laboratory mill (0.5 mm screen). The amount of acid unhydrolyzable residue (AUR, also known as Klason lignin) in the samples was estimated by means of gravimetry, using hot sulfuric acid digestion [15]. The content of reducing sugars was estimated using the phenol–sulfuric acid method [16] to calculate the amount of total carbohydrate (TCH). Total N concentration was measured using a combustion method with an automatic gas chromatograph (NC analyzer SUMIGRAPH NC-900, Sumitomo Chemical, Osaka, Japan). Details of the procedures have been described previously [11]. The mass loss of AUR and TCH was determined as a percentage of the original mass, taking the mass loss of AUR or TCH in the uninoculated and incubated control treatment into consideration. Initial AUR content ranged from 274 to 316 mg/g, TCH content ranged from 581 to 636 mg/g, and total N content ranged from 2.0 to 3.4 mg/g (Table 2).

### 2.4. Statistical Analysis

The AUR/wood mass (AUR/wood) loss ratio and AUR/TCH mass (AUR/TCH) loss ratio are useful indices of the selective delignification caused by each fungal species [4]. The AUR/wood loss ratio and AUR/TCH loss ratio of each fungal species for each tree species of wood were calculated according to the equation:

AUR/wood loss ratio = mass loss of AUR (% of original AUR mass)/mass loss of wood (% of original wood mass)

AUR/TCH loss ratio = mass loss of AUR (% of original AUR mass)/mass loss of TCH (% of original TCH mass)

I used linear mixed-effects models (LMMs) to examine the effects of the origin of isolates and tree species of wood on the mass loss of wood, AUR, and TCH, AUR/wood loss ratio, and AUR/TCH loss ratio, including fungal isolates as the random effect to account for the nested structure. I used R software for Macintosh 3.2.2 (http://www.r-project.org/), with the “lme4” package for the analyses, the “lmerTest” package for *p*-values, and the “multcomp” package for multiple comparisons with Tukey’s test. Regression analyses were conducted for linear relationships between mass loss values and initial chemical composition. Mass loss values were arcsin-transformed because the data were in the form of proportion.

## 3. Results

### 3.1. Mass Loss of Wood and Organic Chemical Components

**Figure 1:**
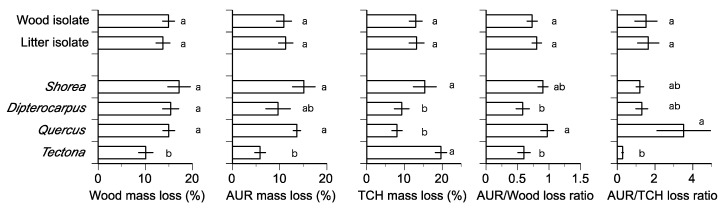
Mean values of mass loss for wood, acid unhydrolyzable residue (AUR), and total carbohydrates (TCH), AUR/wood loss ratio, and AUR/TCH loss ratio. The same letters indicate that there is not a significant difference at the 5% level by Tukey’s HSD test. Bars indicate standard errors.

The mass loss of wood caused by nine *Xylaria* isolates ranged from 4.5% to 28.4% of the original wood mass, and the largest mean mass loss was caused by litter isolate TP5BS101, followed by two wood isolates, TP041004 and TP041001, and the lowest mean mass loss was caused by a litter isolate, TP041009, and a wood isolate, TC041107 (Table 3) [11]. The mean values of the mass loss of wood were not significantly different between wood and litter isolates (LMM, *F* value = 0.193, *p* = 0.6707) and were significantly greater in *Shorea*, *Dipterocarpus*, and *Quercus* than in *Tectona* (LMM, *F* value = 7.869, *p* = 0.0006) (Figure 1).

The mass loss of AUR caused by nine *Xylaria* isolates ranged from 2.5% to 29.8% of the original AUR mass, and the largest mean mass loss was caused by a litter isolate, TP5BS101, followed by two wood isolates, TP041010 and TP041001, and the lowest mean mass loss was caused by a wood isolate, TC041107, and a litter isolate, TP041009 (Table 3). The mean values of mass loss of AUR were not significantly different between wood and litter isolates (LMM, *F* value = 0.026, *p* = 0.8745) and were significantly greater in *Shorea* and *Quercus* than in *Tectona* (LMM, *F* value = 6.982, *p* = 0.0012) (Figure 1).

The mass loss of TCH caused by nine *Xylaria* isolates ranged from 1.0% to 28.6% of the original TCH mass, and the largest mean mass loss was caused by a litter isolate, TP5BS101, and a wood isolate, TP041004, and the lowest mean mass loss by a wood isolate, TC041107 (Table 3). The mean values of mass loss of TCH were not significantly different between wood and litter isolates (LMM, *F* value = 0.006, *p* = 0.9402) and were significantly greater in *Shorea* and *Tectona* than in *Dipterocarpus* and *Quercus* (LMM, *F* value = 12.016, *p* = 0.00004) (Figure 1).

### 3.2. Degree of Selective Delignification

The AUR/wood loss ratio of nine *Xylaria* isolates ranged from 0.28 to 1.53, and the mean values were generally similar between the isolates, except that the lowest value was found for TP041004 (Table 3). The mean values of the AUR/wood loss ratio were not significantly different between wood and litter isolates (LMM, *F* value = 0.6539, *p* = 0.4240) and were significantly greater in *Quercus* than in *Dipterocarpus* and *Tectona* (LMM, *F* value = 5.0740, *p* = 0.0049) (Figure 1).

The AUR/TCH loss ratio of nine *Xylaria* isolates ranged from 0.15 to 12.40, and the largest mean value was found for a wood isolate, TC041107, and the lowest mean mass loss for a wood isolate, TP041004 (Table 3). The mean values of the AUR/TCH loss ratio were not significantly different between wood and litter isolates (LMM, *F* value = 0.0305, *p* = 0.8623) and were significantly greater in *Quercus* than in *Tectona* (LMM, *F* value = 3.9726, *p* = 0.0152) (Figure 1).

### 3.3. Effects of Initial Chemical Composition of Wood

To examine the effect of the chemical differences of wood in tree species, regression analyses were performed for linear relationships between the fungal decomposition and initial chemical composition of wood. The mass loss of wood was significantly and negatively correlated with the initial AUR content of wood (*R*^2^ = 0.193, *n* = 36, *p* = 0.0073) and initial TCH content (*R*^2^ = 0.119, *n* = 36, *p* = 0.0392). The mass loss of AUR was significantly and negatively correlated with the initial AUR content (*R*^2^ = 0.259, *n* = 36, *p* = 0.0015). The mass loss of TCH was significantly and positively correlated with initial AUR content (*R*^2^ = 0.236, *n* = 36, *p* = 0.0027), initial TCH content (*R^2^* = 0.358, *n* = 36, *p* = 0.0001), and initial total N content (*R*^2^ = 0.213, *n* = 36, *p* = 0.0046). The AUR/wood loss ratio and AUR/TCH loss ratio were significantly and negatively correlated with initial AUR content (AUR/wood loss ratio: *R*^2^ = 0.137, *n* = 36, *p* = 0.0265; AUR/TCH loss ratio: *R*^2^ = 0.123, *n* = 36, *p* = 0.0358).

## 4. Discussion

The present study demonstrated that the tropical *Xylaria* isolates examined have a potential ability to cause a mass loss of wood and decompose AUR and TCH in the wood of tropical tree species. Although few data have been available in the previous literature on the AUR decomposition by *Xylaria* species, the values of the mass loss of wood, AUR, and TCH caused by the tropical *Xylaria* isolates (Table 3) are within the range reported for temperate wood decay fungi [7,8,17]. Both wood and litter isolates exhibited a similar ability of wood decomposition, regardless of the original substrata (i.e., wood or litter) from which the isolates were obtained (Figure 1), but this is not surprising as the same fungal species can occur as wood and litter isolates (Table 1) and the distinction between these two ecological groups is somewhat unclear. The production of ligninolytic and cellulolytic enzymes is responsible for the decomposition of wood and wood components by tropical *Xylaria* isolates [5,6,9,12].

The AUR/wood loss ratio was less than 0.8 in 21 (58%) of the 36 cases and was between 0.8 and 1.5 in 14 (39%) (Table 3), indicating that the tropical *Xylaria* isolates in most cases caused a selective decomposition of components other than AURs and also caused the simultaneous decomposition of AUR and other components [7]. A previous study reported a similar value for the AUR/wood loss ratio of 0.5 for *Xylaria polymorpha* inoculated with birch wood [7]. The finding that tropical *Xylaria* species caused the selective decomposition of wood components other than AUR is in accord with previous findings that xylariaceous fungi cause soft-rot-type decomposition by forming cavities within the secondary wall along the microfibrillar axis or cell wall erosion towards the middle lamella [18], in which carbohydrates were preferentially attacked over lignin [19]. In fact, a previous study showed that the ability of *Xylaria* species to cause AUR decomposition is the lowest when compared with other ligninolytic fungal species in such genera as *Mycena*, *Gymnopus*, and *Marasmius* [20].

The mass loss of AUR and TCH caused by the tropical *Xylaria* isolates varied with the tree species of the wood (Figure 1) and was related to the initial chemical composition of wood. The mass loss of wood and AUR, AUR/wood loss ratio, and AUR/TCH loss ratio were negatively related to the initial AUR content of wood, implying the limiting effect of AUR on wood decomposition by *Xylaria* isolates. In fact, the AUR fraction consists of recalcitrant organic compounds, such as tannins, cutins, and lignin [16]. Previous pure culture tests repeatedly demonstrated that the fungal decomposition was negatively correlated with AUR content [4]. In contrast, the mass loss of TCH was positively related not only to the initial contents of organic chemical components but also to initial total N content of wood. The result appears reasonable as nitrogen is known to stimulate the consumption of carbohydrates in wood by fungi [21].

In conclusion, the present study showed that the tropical *Xylaria* isolates had the ability to decompose lignin (as AUR) and carbohydrates in wood and preferred carbohydrates to lignin. To the knowledge of the author, this is the first report on the decomposition of organic chemical components in wood by tropical *Xylaria* species. However, I should note a limitation of the present study, namely, that I used nine *Xylaria* isolates obtained from northern Thailand. *Xylaria* species are well represented in the tropics, and tropical *Xylaria* species are estimated to be diverse in their taxonomic richness, functioning, and host relations [5,6]. Likewise, the wood of four tree species was used as substrata, despite the wealth of tropical tree richness [22]. Future research should include combinations of more fungal and tree species from different localities in pure culture tests to draw comprehensive pictures of the decomposition of organic chemical components by *Xylaria* species.

## Figures and Tables

**Table 1:** *Xylaria* isolates used in the test. UNITE species hypothesis (SH) at 1.5% threshold is indicated [14].

Strain Code	DDBJ Accession Number	UNITE Taxon	UNITE SH Code
**Wood isolate**			
TP041001	AB524021	Xylariaceae sp.	SH1541119.08FU
TP041004	AB524022	Xylariaceae sp.	SH1541152.08FU
TP041010	AB524023	*Xylaria* sp.	SH1541195.08FU
TC041101	AB524024	*Xylaria polymorpha*	SH1541249.08FU
TC041107	AB524025	*Xylaria grammica*	SH1541130.08FU
**Litter isolate**			
TP5BS72	AB524026	Xylariaceae sp.	SH1541152.08FU
TP5BS101	AB524027	Xylariaceae sp.	SH1541119.08FU
TC041102	AB524028	*Xylaria* sp.	SH1522725.08FU
TP041009	AB524029	*Xylaria* sp.	SH1541307.08FU

**Table 2:** Contents (mg/g) of organic chemical components and total nitrogen (N) in wood of four tropical tree species used in pure culture decomposition tests. AUR, acid unhydrolyzable residue; TCH, total carbohydrates.

Tree Species of Wood	AUR	TCH	Total N
*Shorea obtusa*	275	606	3.4
*Dipterocarpus tuberculatus*	282	581	2.0
*Quercus kingiana*	274	589	2.8
*Tectona grandis*	316	636	3.3

**Table 3:** Mass loss (% original mass) of wood, acid unhydrolyzable residue (AUR), and total carbohydrates (TCHs), AUR/wood loss ratio, and AUR/TCH loss ratio after 12 weeks at 20 °C when inoculated with *Xylaria* isolates in vitro. Values of wood mass loss are means with standard errors in parentheses. Data of mass loss of wood were from [11].

	Source	Wood		AUR	TCH	AUR/Wood Loss Ratio	AUR/TCH Loss Ratio
***Shorea obtusa***							
TP041001	Wood	18.1	(1.7)	11.5	14.2	0.63	0.81
TP041004	Wood	28.4	(2.2)	18.5	28.6	0.65	0.65
TP041010	Wood	21.8	(0.7)	29.8	21.9	1.37	1.36
TC041101	Wood	21.2	(0.3)	20.1	16.1	0.95	1.24
TC041107	Wood	8.8	(0.3)	7.8	4.5	0.89	1.74
TP5BS72	Litter	18.8	(1.9)	15.2	14.5	0.81	1.05
TP5BS101	Litter	20.7	(3.1)	16.0	26.8	0.77	0.60
TC041102	Litter	8.8	(0.7)	10.2	4.2	1.17	2.44
TP041009	Litter	7.8	(0.8)	7.0	7.6	0.90	0.92
***Dipterocarpus tuberculatus***							
TP041001	Wood	18.3	(1.1)	22.1	12.0	1.21	1.85
TP041004	Wood	17.4	(0.6)	5.3	12.7	0.31	0.42
TP041010	Wood	13.7	(0.5)	6.0	4.0	0.44	1.51
TC041101	Wood	16.4	(1.1)	4.9	4.2	0.30	1.18
TC041107	Wood	10.2	(0.3)	2.8	4.3	0.28	0.66
TP5BS72	Litter	14.1	(1.0)	9.0	2.7	0.64	3.37
TP5BS101	Litter	25.9	(0.9)	23.6	15.8	0.91	1.49
TC041102	Litter	13.9	(1.8)	9.2	9.3	0.66	0.99
TP041009	Litter	8.6	(0.4)	4.2	18.3	0.48	0.23
***Quercus kingiana***							
TP041001	Wood	19.6	(5.4)	13.5	10.2	0.69	1.32
TP041004	Wood	17.7	(0.3)	10.3	11.4	0.58	0.90
TP041010	Wood	13.3	(1.9)	13.5	8.0	1.01	1.69
TC041101	Wood	16.2	(2.4)	11.9	9.5	0.74	1.25
TC041107	Wood	9.0	(1.3)	11.8	1.0	1.32	12.40
TP5BS72	Litter	14.3	(0.5)	15.1	12.3	1.05	1.22
TP5BS101	Litter	18.4	(0.7)	18.4	8.7	1.00	2.12
TC041102	Litter	16.5	(1.0)	13.6	9.4	0.83	1.45
TP041009	Litter	9.8	(0.4)	14.9	1.6	1.53	9.32
***Tectona grandis***							
TP041001	Wood	11.8	(3.3)	7.0	20.0	0.59	0.35
TP041004	Wood	11.3	(1.0)	5.7	24.2	0.51	0.24
TP041010	Wood	12.2	(0.7)	6.7	21.6	0.55	0.31
TC041101	Wood	4.8	(0.7)	6.3	16.3	1.30	0.38
TC041107	Wood	8.5	(0.3)	2.8	14.9	0.33	0.19
TP5BS72	Litter	10.3	(1.9)	5.7	19.2	0.56	0.30
TP5BS101	Litter	19.4	(1.3)	13.6	28.4	0.70	0.48
TC041102	Litter	8.0	(0.7)	2.5	16.6	0.31	0.15
TP041009	Litter	4.5	(0.4)	2.6	15.7	0.58	0.17

## References

[1] Boddy L., Frankland J.C., van West P. (2008). Ecology of Saprotrophic Basidiomycetes.

[2] Baldrian P. (2017). Forest microbiome: Diversity, complexity and dynamics. FEMS Microbiol. Rev..

[3] Eriksson K.E., Blanchette R.A., Ander P. (1990). Microbial and Enzymatic Degradation of Wood and Wood Components.

[4] Osono T. (2020). Functional diversity of ligninolytic fungi associated with leaf litter decomposition. Ecol. Res..

[5] Whalley A.J.S. (1996). The xylariaceous way of life. Mycol. Res..

[6] Rogers J.D. (2000). Thoughts and musings on tropical Xylariaceae. Mycol. Res..

[7] Worrall J.J., Anagnost S.E., Zabel R.A. (1997). Comparison of wood decay among diverse lignicolous fungi. Mycologia.

[8] Fukasawa Y., Osono T., Takeda H. (2005). Decomposition of Japanese beech wood by diverse fungi isolated from a cool temperate deciduous forest. Mycoscience.

[9] Pointing S.B., Parungao M.M., Hyde K.D. (2003). Production of wood decay enzymes, mass loss and lignin solubilization in woody by tropical Xylariaceae. Mycol. Res..

[10] Pointing S.B., Pelling A.L., Smith G.J.D., Hyde K.D., Reddy C.A. (2005). Screening of basidiomycetes and xylariaceous fungi for lignin peroxidase and laccase gene-specific sequences. Mycol. Res..

[11] Osono T., To-Anun C., Hagiwara Y., Hirose D. (2011). Decomposition of wood, petiole, and leaf litter by *Xylaria* species from northern Thailand. Fungal Ecol..

[12] Liers C., Arnstadt T., Ullrich R., Hofrichter M. (2011). Patterns of lignin degradation and oxidative enzyme secretion by different wood- and litter-colonizing basidiomycetes and ascomycetes grown on beech-wood. FEMS Microbiol. Ecol..

[13] Preston C.M., Trofymow J.A., Sayer B.G., Niu J. (1997). ^13^C nuclear magnetic resonance spectroscopy with cross-polarization and magic-angle spinning investigation of the proximate-analysis fractions used to assess litter quality in decomposition studies. Can. J. Bot..

[14] Nilsson R.H., Larsson K.H., Taylor A.F.S., Bengtsson-Palme J., Jeppesen T.S., Schigel D., Kennedy P., Picard K., Glöckner F.O., Tedersoo L. (2019). The UNITE database for molecular identification of fungi: Handling dark taxa and parallel taxonomic classifications. Nucleic Acids Res..

[15] King H.G.C., Heath G.W. (1967). The chemical analysis of small samples of leaf material and the relationship between the disappearance and composition of leaves. Pedobiologia.

[16] Dubois M., Gilles K.A., Hamilton J.K., Rebers P.A., Smith F. (1956). Colorimetric method for determination of sugars and related substances. Anal. Chem..

[17] Tanesaka E., Masuda H., Kinugawa K. (1993). Wood degrading ability of basidiomycetes that are wood decomposers, litter decomposers, or mycorrhizal symbionts. Mycologia.

[18] Blanchette R.A. (1995). Degradation of the lignocellulose complex in wood. Can. J. Bot..

[19] Nilsson T., Daniel G. (1989). Chemistry and microscopy of wood decay by some higher ascomycetes. Holzforschung.

[20] Osono T. (2015). Decomposing ability of diverse litter-decomposer macrofungi in subtropical, temperate, and subalpine forests. J. For. Res..

[21] Reid I.D. (1991). Nutritional regulation of synthetic lignin (DHP) degradation by *Phlebia* (*Merulius*) *tremellosa*: Effect of nitrogen. Can. J. Bot..

[22] Corlett R.T. (2009). The Ecology of Tropical East Asia.

